# Working Men and Representation

**Published:** 1914-07-25

**Authors:** 


### WORKING MEN AND REPRESENTATION.

### Mr. Roden Orde on the Principle and its Application.

The Royal Victoria Infirmary, Newcastle, which many of our readers have recently visited in connection with the annual Conference of the British Hospitals Association in June, stands out as one of the two institutions in the country where working men's contributions have reached a total of over £20,000 a year. Now that contributions from the industrial classes are spreading in those districts not primarily manufacturing, where the shop takes the place of the factory, and the southern industrial cities are beginning to follow those in the north in the organisation of working men's collections on a large scale, the experience of those institutions in which representation has been existing for some time past becomes of special interest. Our Commissioner therefore visited Mr. Roden Orde, the house governor and secretary of the Royal Victoria Infirmary, Newcastle, to learn from him the lessons which had been gained on the subject at his institution.

### Representation as a Right.

"Is representation regarded as the necessary right of giving a subscription?"

"It is," Mr. Orde replied, "and as under this hospital's constitution a subscription of only £10 carries with it the right to a governorship, the number of governors is very large. Our system here is to divide the locality into areas of working men, each of which meets once a year in order to elect its representatives. These representatives are then nominated and sent up to the whole body of governors, which thereupon elects the governing board. A third of this board is composed of representatives of the ordinary two-guinea subscribers, a third of representatives of working men, and the remainder of consulting physicians and the staff. The startling result is that the board of management now consists of actually sixty-five persons, nor does there appear any reason (except the practical disadvantage which many would not recognise as a reason at all) to suppose that the limit has been reached. For instance, till two years ago the number of working men's representatives was sixteen, but at that time it was raised to twenty-four on account of the increased support that was coming from this quarter."

"This shows the tendency?"

"I am afraid that it does. And I say afraid because, as everyone knows, the larger the committee the more difficult does it become to expedite business, till, as it seems, the last stage is reached in a huge assembly like the House of Commons, in which, owing to the practical difficulty of getting anything done, a variety of expedients like the closure are resorted to, and the assembly which began with an intention of representing everybody develops into a machine for registering the decisions of a majority, which itself may represent nothing but the chance faction which the opposition of interests throws for the time into the saddle. There is no doubt that the working man has at the back of his mind the feeling that if he subscribes he has a claim to representation and a claim to treatment; and the attitude of the employers is that as he benefits so he should contribute. Recent legislation has helped this idea, with the result that the employer does not realise to what extent—and it is a very large one—the hospital benefits him; and the working man does not realise how small a proportion even his contributions bear to the cost of the treatment which he receives."

### The Delegate's Dilemma.

"How does the claim for representation affect the representatives chosen?"

"A representative is elected first of all as a delegate of the group which appoints him. He begins, at their behest, to attend committee meetings where, if he is an able man, as is frequently the case, his eyes soon open to the fact that hospital management is a technical and complicated business, and that the good of the hospital may be actually imperilled by the proposals of this or that section of subscribers who are unacquainted with its internal administrative needs. In other words, our delegate's education is proceeding, the fascination of the work and the desire, born of experience, to secure the best result for the *institution*, are making their mark upon him. But those who have appointed him have not shared his experience, and frequently are extremely slow, having no responsibility of management, to modify preconceived ideas. What is the result? It is, sooner or later, that the delegate acts in the light of experience instead of at the dictation of those who appoint him. Friction may follow. He may be reminded that he was elected to carry out instructions, accused of repudiating his pledges, and, at the next opportunity, he may be turned out. From the point of view of the hospital, this means that the same educative process has to be gone through all over again with someone else. The representative then is placed in a very difficult position, and, as I once ventured to sum up the moral in an address, while the governors are quite right in reminding their delegates that the men who pay the piper have the right to call the tune, this right does not include that of playing the instrument. It is difficult to drive this home without being accused of class-prejudice, but it applies to every class equally. Brains and energy are thrown about in as haphazard a fashion as anything else in Nature, and education in management is necessary to all."

### "Interference is not Management."

"The claim for representation affects the constitution of the committees?"

"Yes, and not only as regards the individual representatives who compose them. The difficulties of such men I have already pointed out; but there is also the effect of numbers. If so low a sum as £10 is fixed on as giving the right to a governorship, this means that the committees are far too large for management purposes. When you have committees running up to twenty-five, forty, even seventy, the committee has developed into a debating society, and has defeated its own object by making management almost impossible. It is, unfortunately, extremely difficult to get people to realise that interference is not management, and that democratic control does not mean a multitude of counsellors. Yet any student who has devoted thought to the subject will tell you that democracy itself does not mean 'government by the people for the people,' but government by *consent of* the people, which is a very different thing. It means that executives are elected by the people, and that they are given a free hand, with the knowledge that at the end of a defined interval the executive will have to give an account of their stewardship, and can be turned out if those whose representatives they are do not feel satisfied with the result. One has often heard it said that a committee of one is the best of all committees, and provided that that one is held fully responsible for his actions and is appointed by a majority for a limited term there is something to be said for this view. Interference is not management : that is the truth."

### Medical Men on Management Committees.

"How about the representation of the honorary medical staff?"

"They are typical of other interests, and provide a good instance of the way in which it is desirable to have a channel of intercommunication between the medical and administrative sides of a hospital. Causes of misunderstanding are removed by such a means, and if one observes the cases of friction which do arise from time to time in different parts of the country they seem to appear where no recognised channel of this kind exists. I wish rather to consider, however, the question of representation, not by itself, but as it affects management; and in the case of the honorary medical staff this is what we find. Management is not interesting to most medical men, and you get eminent physicians like Sir William Osler boasting that 'there is probably no one so long and intimately associated with boards of management who really knows less about their administration. . . . I have persistently avoided knowing anything about administration as such.' So we see that the only point of importance to consider is not whether the honorary staff should or should not have a voice in the management of this hospital, but how this voice is to be best made heard. It is at least open to question whether direct representation is the best method of affecting it. The medical staff are too busy, even if they were individually inclined, to attend regularly. The result is that they do not attend except when their own interests are involved. When this is the case there is a danger that they may vote not as managers but as delegates; and in order to become conversant with the point of view of a manager—that is to say of a man who has the best interests of the hospital solely in view—it is essential to attend committee meetings with regularity. Questions of management must be treated as a whole—individual interests have sometimes to be subordinated for this purpose."

### The Origin of the Workmen's Fund.

"What was the origin of your workmen's subscriptions here?"

"The subscriptions started at the Elswick Works in the Scotswood Road, Newcastle-upon-Tyne, where the Armstrong-Whitworth guns are made, and originated out of the desire of the men to contribute something to the hospital. But I believe that Newcastle was not the first centre to start them. Sunderland Infirmary, I believe, preceded us in the matter. The idea of helping the hospital was of spontaneous growth. Collections at the works were organised, other firms followed suit, and the movement has spread so that now mines and many other industries are included in it. If it had been realised how widely this movement would grow, possibly a distinction would have been drawn between the amount of an individual donation which should carry a governorship with it and that collected by a body of persons, or else the qualifying sum should have been raised all round. It is obvious that £10 is far too small to carry the right of governorship with it : £50 or £100 would be nearer the mark. Such rights must naturally develop as the institution with which they are associated; and it is manifest that £10 bore a very different proportion to the Royal Victoria Infirmary's income and expenditure at the time when a donation of this amount carried a governorship with it than it does to-day. The result of not raising it is that there are too many governors; too large committees; too much interference; delays, and a general tendency to make management the plaything of a number."

"Is it impossible to raise the qualification?"

"Any proposal to do so would be extremely unpopular, and would be opposed. The suggestion would be made that there was a wish to deprive the workmen of their rights, and it would be difficult to convince them that an overgrown committee has defeated its own object. Numbers, again, affect the sub-committees. For instance, we have a nursing staff committee of twenty-seven—nearly half the total committee; and for this particular purpose it is seriously open to question whether there should be a sub-committee at all. The selection of nurses and probationers is really work for one person only. Such points cannot be decided by a number of men without risk of undue influence in many ways A large committee is not a place in which to discuss individual qualifications, or the inner reasons which may determine the institution's decision in respect of this or that candidate. A large committee is a place where pressure may be brought by individual members on the matron to appoint particular probationers or nurses, when the decision should rest upon her judgment alone. There is a tendency in this multitude of counsellors to undermine discipline; and while actual trouble seldom arises, there is the constant anxiety lest it should do so."

### Representation of other Classes.

"How about the representation of other classes of givers besides working men?"

"Other classes do not bother about representation, and do not concern themselves in matters of management. This is the root of the whole question. As a matter of fact the representation of the working classes is very great, much greater in proportion than that of other classes. The vital importance of the voluntary hospital is felt more immediately by the working classes than by any others; other classes only feel the benefit indirectly, and do not understand how much they really owe. The attendance at courts of governors illustrates this. In London and in the south generally these courts are a farce—hospitals there are faced with such problems as waking up the annual meeting and the like—here they are an event of the first magnitude. Another side of this is seen in the case of employers' subscriptions. There used to be a personal relation between the employer and his men, and his subscription to the hospital was a personal matter. Now with the growth of the joint-stock company hospital subscriptions become a resolution of the board, and, curiously enough, are sometimes withdrawn on the excuse of recent legislation, even when the new enactments have been supported by the directors."

### Employers' Subscriptions and Compensation Cases.

"Do employees realise the value of the hospital to them?"

"I think not, though it is very easy to show. Take, for example, our waiting list. We have an absolute waiting list of nearly 1,000, many of which are compensation cases. Now the employer says, "I have already had to pay so much in compensation, and so I fail to see why I should increase my losses by subscribing to a hospital." The truth is that without the institution the employers would have to pay far more. For the hospital gets the man to whom compensation is being paid back to work at the earliest possible moment. Take, for instance, an accident case. Compensation is paid to the injured man. He gets half his wages, and the employer receives no work in return. So long as the man is receiving compensation he is a dead loss to the employer. During the time when compensation is being paid the man is on our waiting list, where he may remain six or nine months. The compensation paid to him for this period amounts to a considerable sum. Had we more beds or were there another hospital in Newcastle the waiting list problem would be lessened, and the man got back to work in probably a tenth of the time. Even as things are, it would in many cases cost the employer less to send the injured man to a private home, to pay, say, £15 a case, and to get him back at work again in a fortnight.

A Chance for a Pay Hospital.

" In fact, there exists an opportunity for a pay hospital on these lines of 100 beds in Newcastle to take patients at a fee of £15 a case. As I have said, it would pay the employers to pay this sum for a speedy recovery. The waiting-list is a problem in all the voluntary hospitals, and further accommodation is required, but the voluntary system does not seem to me to be capable of finding the necessary capital sums. An advance of £100,000 is needed, on which the hospital would have to pay interest, and a sinking fund. If the voluntary system can be provided with the necessary capital sums for the provision of further accommodation, it can go on and pay its maintenance sums. As to further accommodation, not only beds but the size of institutions has to be considered. My own opinion is that it is better to have two hospitals of 500 than one of 1,000. The State could make the capital grants required."

" How about the doctors were these grants forthcoming?"

" Grants in aid are a very different thing from State support. Contributions by patients have not meant paid doctors. Again, already the State makes grants to educational establishments without insisting on any real control, and such inspection as it does insist on is, I understand, perfectly unobjectionable. As public opinion is very much open to influence at the present time, so far as hospitals are concerned, I hope those who are best informed may mould public opinion so as to induce the State to grant her aid without depriving the hospitals of their freedom of management. There seems no very good reason why some halfway house should not be found in which the State, by bearing the financial burden, relieves the managers of the danger of allowing their policy to be dictated by any other consideration than the good of the hospital. For this question of finance, and the accommodation which hangs upon it, is of the very essence of the representation problem. It may be thought that pay-wards at this institution might be suggested. But they would not be popular here, as several of the staff have nursing homes of their own.

The value of a hospital of 500 beds to the community can easily be calculated in terms of money—it is more than £100,000 a year. This is a fact which should not be forgotten by those who imagine they are giving more than they get.

